# Fatality rate and predictors of mortality in an Italian cohort of hospitalized COVID-19 patients

**DOI:** 10.1038/s41598-020-77698-4

**Published:** 2020-11-26

**Authors:** Mattia Bellan, Giuseppe Patti, Eyal Hayden, Danila Azzolina, Mario Pirisi, Antonio Acquaviva, Gianluca Aimaretti, Paolo Aluffi Valletti, Roberto Angilletta, Roberto Arioli, Gian Carlo Avanzi, Gianluca Avino, Piero Emilio Balbo, Giulia Baldon, Francesca Baorda, Emanuela Barbero, Alessio Baricich, Michela Barini, Francesco Barone-Adesi, Sofia Battistini, Michela Beltrame, Matteo Bertoli, Stephanie Bertolin, Marinella Bertolotti, Marta Betti, Flavio Bobbio, Paolo Boffano, Lucio Boglione, Silvio Borrè, Matteo Brucoli, Elisa Calzaducca, Edoardo Cammarata, Vincenzo Cantaluppi, Roberto Cantello, Andrea Capponi, Alessandro Carriero, Francesco Giuseppe Casciaro, Luigi Mario Castello, Federico Ceruti, Guido Chichino, Emilio Chirico, Carlo Cisari, Micol Giulia Cittone, Crizia Colombo, Cristoforo Comi, Eleonora Croce, Tommaso Daffara, Pietro Danna, Francesco Della Corte, Simona De Vecchi, Umberto Dianzani, Davide Di Benedetto, Elia Esposto, Fabrizio Faggiano, Zeno Falaschi, Daniela Ferrante, Alice Ferrero, Ileana Gagliardi, Gianluca Gaidano, Alessandra Galbiati, Silvia Gallo, Pietro Luigi Garavelli, Clara Ada Gardino, Massimiliano Garzaro, Maria Luisa Gastaldello, Francesco Gavelli, Alessandra Gennari, Greta Maria Giacomini, Irene Giacone, Valentina Giai Via, Francesca Giolitti, Laura Cristina Gironi, Carla Gramaglia, Leonardo Grisafi, Ilaria Inserra, Marco Invernizzi, Marco Krengli, Emanuela Labella, Irene Cecilia Landi, Raffaella Landi, Ilaria Leone, Veronica Lio, Luca Lorenzini, Antonio Maconi, Mario Malerba, Giulia Francesca Manfredi, Maria Martelli, Letizia Marzari, Paolo Marzullo, Marco Mennuni, Claudia Montabone, Umberto Morosini, Marco Mussa, Ilaria Nerici, Alessandro Nuzzo, Carlo Olivieri, Samuel Alberto Padelli, Massimiliano Panella, Andrea Parisini, Alessio Paschè, Alberto Pau, Anita Rebecca Pedrinelli, Ilaria Percivale, Roberta Re, Cristina Rigamonti, Eleonora Rizzi, Andrea Rognoni, Annalisa Roveta, Luigia Salamina, Matteo Santagostino, Massimo Saraceno, Paola Savoia, Marco Sciarra, Andrea Schimmenti, Lorenza Scotti, Enrico Spinoni, Carlo Smirne, Vanessa Tarantino, Paolo Amedeo Tillio, Rosanna Vaschetto, Veronica Vassia, Domenico Zagaria, Elisa Zavattaro, Patrizia Zeppegno, Francesca Zottarelli, Pier Paolo Sainaghi

**Affiliations:** 1 Department of Translational Medicine, Università del Piemonte Orientale UPO, Via Solaroli 17, 28100 Novara, NO Italy; 2 Azienda Ospedaliero Universitaria “Maggiore Della Carita”, Novara, Italy; 3 Presidio Ospedaliero S. Andrea, ASL VC, Vercelli, Italy; 4 Azienda Ospedaliera SS. Antonio E Biagio E Cesare Arrigo, Alessandria, Italy

**Keywords:** Viral infection, Risk factors

## Abstract

Clinical features and natural history of coronavirus disease 2019 (COVID-19) differ widely among different countries and during different phases of the pandemia. Here, we aimed to evaluate the case fatality rate (CFR) and to identify predictors of mortality in a cohort of COVID-19 patients admitted to three hospitals of Northern Italy between March 1 and April 28, 2020. All these patients had a confirmed diagnosis of SARS-CoV-2 infection by molecular methods. During the study period 504/1697 patients died; thus, overall CFR was 29.7%. We looked for predictors of mortality in a subgroup of 486 patients (239 males, 59%; median age 71 years) for whom sufficient clinical data were available at data cut-off. Among the demographic and clinical variables considered, age, a diagnosis of cancer, obesity and current smoking independently predicted mortality. When laboratory data were added to the model in a further subgroup of patients, age, the diagnosis of cancer, and the baseline PaO_2_/FiO_2_ ratio were identified as independent predictors of mortality. In conclusion, the CFR of hospitalized patients in Northern Italy during the ascending phase of the COVID-19 pandemic approached 30%. The identification of mortality predictors might contribute to better stratification of individual patient risk.

## Introduction

Coronavirus disease 2019 (COVID-19) is a pandemic infection caused by SARS-CoV-2; its clinical manifestations encompass a wide range of entities, from a mild flu-like illness to life-threatening forms^1^. The first reports describing the clinical characteristics of patients affected by COVID-19 have been from China, where the pandemic originated at the end of 2019^2,3^; since the first case, reported on 20th February 2020, the outbreak in Italy has rapidly assumed dramatic proportions, making it the first Western country to face this epidemic.

One of the most striking aspects of the data diffused by the World Health Organization (WHO) is the substantially different prognosis among countries; indeed, according to the last situation report (18th May 2020), the case fatality rate (CFR) in Italy (31,908/225,435; 14.15%) is considerably higher than in China (4,645/84,494; 5.5%)^4^. This almost three-fold increased risk of death in Italy has been explained with a higher susceptibility to mortality risk factors: age, male gender, and comorbidities^5^. However, even among Western countries, there are relevant differences: for instance, Germany reported a very low fatality rate (4.54%) when compared to France (20.04%), United Kingdom (14.21%) or Spain (11.95%)^4^. The reasons for these discrepancies are still unclear and may include genetic factors, differences in the local testing strategies, and epidemiological reporting between countries and a different ability of local health systems to deal with the epidemic.

To better elucidate this issue, it is particularly important to have a clear picture of the general features of the patients diagnosed with COVID-19 in different countries. Data about the clinical features of Italian Covid-19 patients admitted to hospital are still lacking. The present study aims to fill this gap.

## Methods

### Study population

The study was conducted in three hospitals in Northern Italy (“Maggiore della Carità” University Hospital in Novara, “Santi Antonio e Biagio e Cesare Arrigo” Hospital in Alessandria and “Sant’Andrea” Hospital in Vercelli). The hospitals are the referral of a vast homogenous territory in Eastern Piedmont, one of the provinces most severely hit by the COVID-19 outbreak, with a catchment area of around 900,000 inhabitants.

From hospital administrative data revision, we selected all consecutive patients older than 18 years of age, admitted to the hospital after Emergency Room evaluation, with a confirmed diagnosis of SARS-CoV-2 infection by reverse-transcriptase polymerase chain reaction (RT-PCR) of a nasopharyngeal swab, between 1st March 2020 and 28th April 2020.

On 9th April 2020, an electronic case report form was generated using the Research Electronic Data Capture software (REDCap, Vanderbilt University) to retrospectively collect clinical data, retrieved from the revision of clinical records. Data entry was performed by clinicians involved in the management of COVID-19 patients. We assigned a unique pseudonymized code to each patient included in the study.

The following information was collected:

- Patients’ demographics, symptoms, comorbidities, home medications, triage vitals, and complications during the hospital stay;
- Outcomes: the outcome of in-hospital stay (discharged or deceased). We defined as adverse outcome to calculate CFR death for any cause occurred during hospital in-stay.

**Figure 1:**
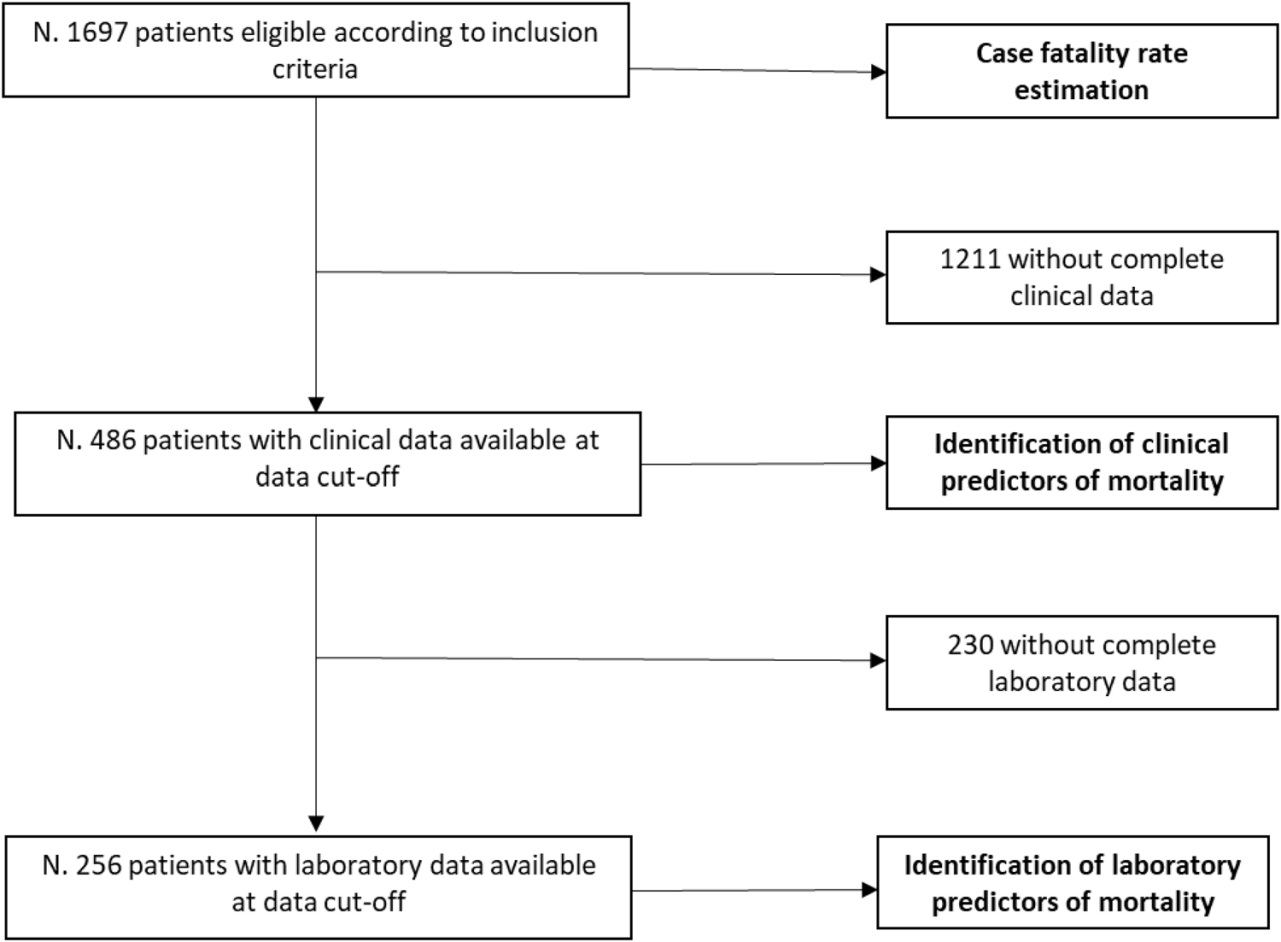
The figure details the selection of the study population.

On 28th April, at the time of cut-off, clinical data about 486/1697 COVID-19 patients admitted during the study period were recorded on the database and were used for the identification of predictors of mortality. Laboratory data were directly retrieved from the central lab system for all the patients admitted to the Novara Hospital (256/486, 53%) and were used to identify the impact on survival (also see Fig. 1 for more details).

The study protocol was approved by the Institutional Review Board (Comitato Etico Interaziendale Novara; IRB code CE 97/20) and conducted in strict accordance with the principles of the Declaration of Helsinki. Prospective informed consent was waived by competent authorities due to the retrospective nature of the study and the use of pseudonymized data (Comitato Etico Interaziendale di Novara).

Patients included in this study have been evaluated for other reports.

### Statistical analysis

Data were summarized according to groups as median and [25th–75th percentile] and analyzed using the Wilcoxon test. Categorical variables, whenever dichotomous or nominal, were reported as frequencies and percentages and analyzed through the Chi-square test.

A univariable logistic regression analysis was carried out to evaluate the effects of covariates on in-hospital mortality.

A random Forest feature selection algorithm was carried out to select the most relevant death predictors to be included in the multivariable logistic regression analysis. The mean decrease in accuracy measure has been considered for computation. The tuning of the algorithm has been performed via a cross-validated procedure with fourfold. The mean decrease in accuracy was considered for variable selection. The algorithm was implemented to select the most relevant variables separately among three different sets of predictors:

1. Set 1. The first set is characterized by the anamnestic predictors.
2. Set 2. The second set includes the laboratory analysis variables.
3. Set 3. The third set is composed of the anamnestic and laboratory analysis predictors.

Three separate logistic regression multivariable analyses were carried out on the different predictors sets considering the relevant features identified by the random forest algorithm. Set 1 was carried out on the population of 486 patients with clinical data recorded; set 2 and 3 were carried out using the 256 subjects for whom laboratory data were available.

The 0.632 bootstrap (1000 resamples) validation procedure was carried out to evaluate the predictive logistic regression model performance reporting the Harrell-C statistics corrected for over-optimism^6^.

The area under curve (AUC) together with the 95% confidence interval has been computed for the multivariable continuous significant predictors.

Statistical analyses were conducted using R 3.5.2^7^ with the RandomForest^8^ and rms^9^ packages. The threshold of statistical significance was 0.05 for all tests used (two-tailed).

## Results

### Analysis of mortality in the study population

We included a total of 1697 patients who were hospitalized because of COVID-19. The fatality rate in this population was 29.7% (504/1697; 95% CI 27.5%; 31.9%). The death incidence over 1000 person-day was 4.9 (95% CI 0.5; 9.3).

**Figure 2:**
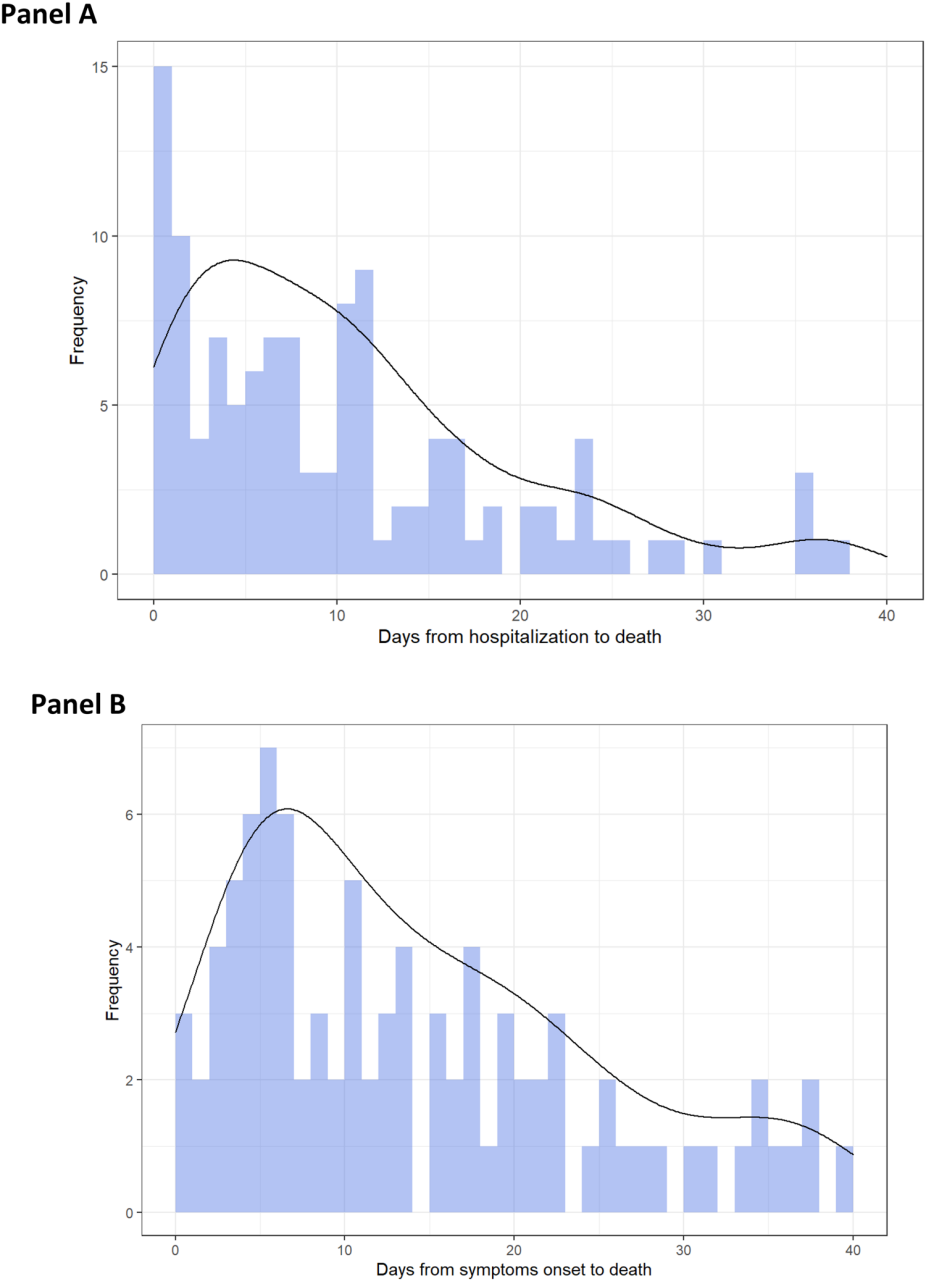
Histogram frequency plot for days until death from hospitalization **(**panel **A**) or symptoms onset **(**panel **B**).

**Figure 3:**
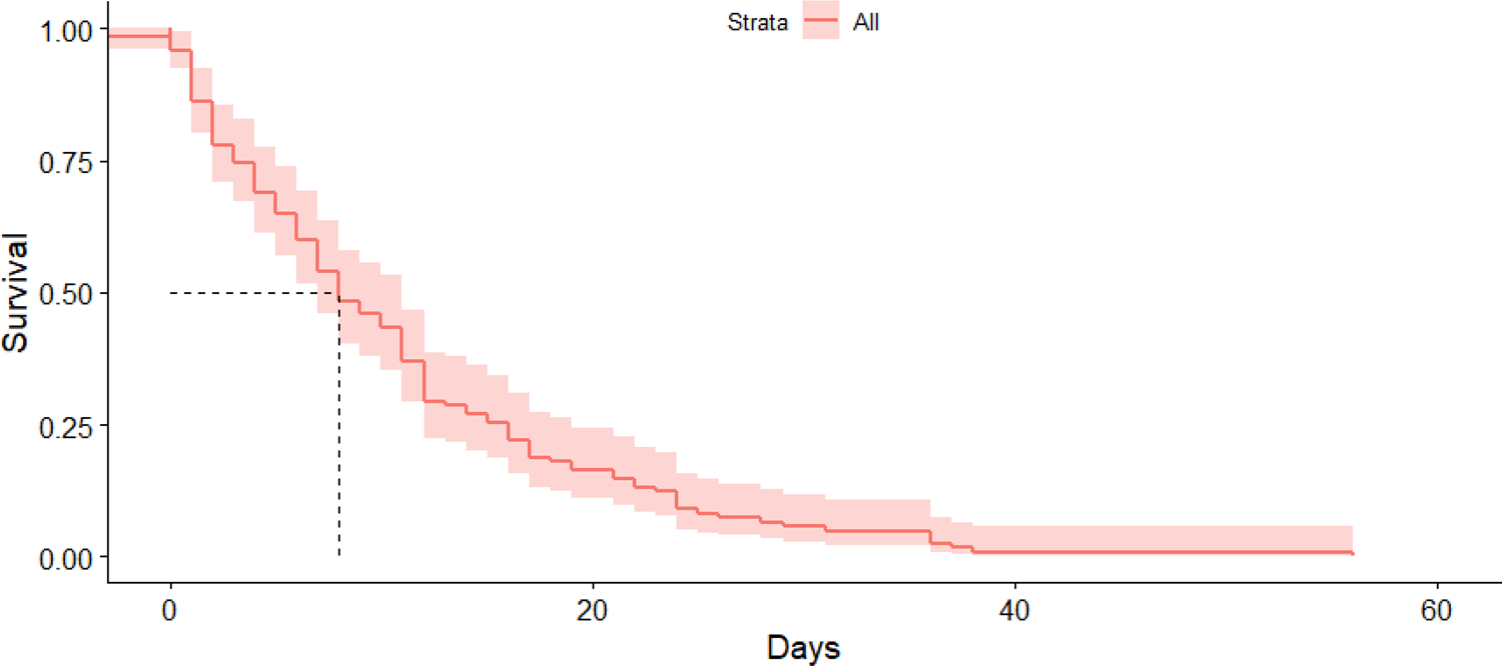
In-hospital survival curve. The median in-hospital survival time is equal to 8 days (95% CI 7; 11).

The median time from symptoms presentation to hospital admission was 5 days^2–9^. In Fig. 2 we report the histogram frequency plot days from hospital admission or symptoms onset. The highest number of deaths are concentrated within the first 12 days of hospital in-stay and 23 days from symptoms onset; however, deaths occurred up to 40 days since hospital admission. In Fig. 3, we report the survival curve for in-hospital mortality; the median in-hospital survival time was 8 days from admission (95% CI 7; 11).

### Clinical predictors of mortality

Predictors of mortality were evaluated in a subset of 486 patients. At the end of the observation period, 407/486 had reached either of the two outcomes (death or discharge), while 79 were still hospitalized; the CFR in this subgroup was 29.9% (122 patients), representative of that observed in the whole population. In Table 1, we report the main clinical features of the study population. The majority were elderly males. Arterial hypertension was the most frequently reported comorbidity. When we looked at the presenting complaints, fever, cough, and dyspnea were the most commonly reported (61%, 59%, and 48% of patients respectively).

**Table 1:** Clinical features of the study population.

	Total (N. 407)	Discharged (N. 285)	Dead (N. 122)	*p*
**Age (years)**	71 [58–80]	65 [54–77]	77 [72–85]	< 0.001
**Gender (M/F)**	239 (59)/168 (41)	165 (58)/120 (42)	74 (61)/48 (39)	0.58
**Comorbidities**				
Arterial Hypertension (y/n)	221 (58)/163 (42)	138 (51)/133 (49)	83 (73)/30 (27)	< 0.001
Diabetes (y/n)	89 (24)/280 (76)	58 (22)/201 (78)	31 (28)/79 (72)	0.23
Smoking (y/n)	54 (17)/258 (83)	30 (14)/191 (86)	24 (26) /67 (74)	0.007
Ischemic cardiopathy (y/n)	60 (16)/306 (84)	32 (12)/225 (88)	28 (26)/81 (74)	0.002
Obesity (y/n)	60 (23)/200 (77)	39 (20)/153 (80)	21 (31)/47 (69)	0.08
COPD (y/n)	37 (10)/329 (90)	23 (9)/238 (91)	14 (13)/91 (87)	0.19
Active Malignancy (y/n)	33 (11)/277 (89)	15 (7)/209 (93)	18 (21)/68 (79)	< 0.001
Chronic liver disease (y/n)	13 (4)/351 (96)	6 (2)/251 (98)	7 (7)/100 (93)	0.05
Autoimmune disease (y/n)	11 (3)/352 (97)	6 (2)/251 (98)	5 (5)/101 (95)	0.23
Atrial fibrillation (y/n)	45 (14)/283 (86)	26 (11)/212 (89)	19 (21)/71 (79)	0.02
Interstitial lung disease (y/n)	10 (3)/318 (97)	6 (3)/231 (97)	4 (4)/87 (96)	0.38
Dementia (y/n)	56 (17)/273 (83)	27 (11)/211 (89)	29 (32)/62 (68)	< 0.001
Chronic kidney disease (y/n)	50 (15)/279 (85)	22 (9)/215 (91)	28 (30)/64 (70)	< 0.001
**Symptoms at presentation**				
Cough (y/n)	209 (55)/168 (45)	160 (59)/109 (41)	49 (45)/59 (55)	0.013
Fever (y/n)	234 (61)/149 (39)	161 (60)/107 (40)	73 (63)/42 (73)	0.53
Myalgia/fatigue (y/n)	112 (31)/254 (69)	85 (33)/175 (67)	27 (25)/79 (75)	0.17
Headache (y/n)	20 (6)/339 (94)	15 (6)/239 (94)	4 (4)/101 (96)	0.67
Hemoptysis (y/n)	5 (1)/356 (99)	3 (1)/253 (99)	2 (2)/103 (98)	0.59
Diarrhea (y/n)	55 (15)/306 (85)	44 (17)/212 (83)	11 (10)/94 (90)	0.11
Dyspnea (y/n)	216 (56)/170 (44)	131 (48)/141 (52)	85 (75)/29 (25)	< 0.001
Chest pain (y/n)	20 (6)/341 (94)	18 (7)/238 (93)	2 (2)/103 (98)	0.06
Sore throat (y/n)	9 (3)/306 (97)	7 (3)/221 (97)	2 (2)/85 (98)	0.71
Anosmia (y/n)	17 (5)/296 (95)	14 (6)/212 (94)	3 (3)/84 (97)	0.34
Upper airway congestion (y/n)	9 (3)/305 (97)	7 (3)/219 (97)	2 (2)/86 (98)	0.69
**Vitals**				
Body temperature (°C)	37.5 [36.5–38.0]	37.4 [36.5–38.0]	37.6 [36.5–38.0]	0.3
Heart rate (per min)	85 [75–98]	84 [74–95]	86 [76–100]	0.12
Respiratory rate (per min)	20 [18–28]	19 [16–23]	26 [22–34]	< 0.001
Systolic blood pressure (mmHg)	124 [115–140]	125 [115–140]	120 [110–140]	0.47
Diastolic blood pressure (mmHg)	70 [68–80]	74 [70–80]	70 [62–80]	0.02

The main general features of study population are reported. Comparison between survivors and dead have been performed by a non-parametric Wilcoxon test or by the Chi-square test, as opportune. For abbreviation: M, males; F, females; y, yes; n, no; COPD, chronic obstructive pulmonary disease.

We then evaluated the prevalence of underlying comorbidities among deceased patients and survivors: arterial hypertension, history of coronary artery disease (CAD), active cancer, atrial fibrillation, dementia and chronic kidney disease were all more prevalent in patients who died during the hospital stay. Similarly, current smoking was positively associated with mortality. Concerning the clinical picture at admission, the patients with a poorer prognosis had a median higher respiratory rate and had more frequently dyspnea.

We further run a univariate analysis which is reported in supplementary Table 1.

**Figure 4:**
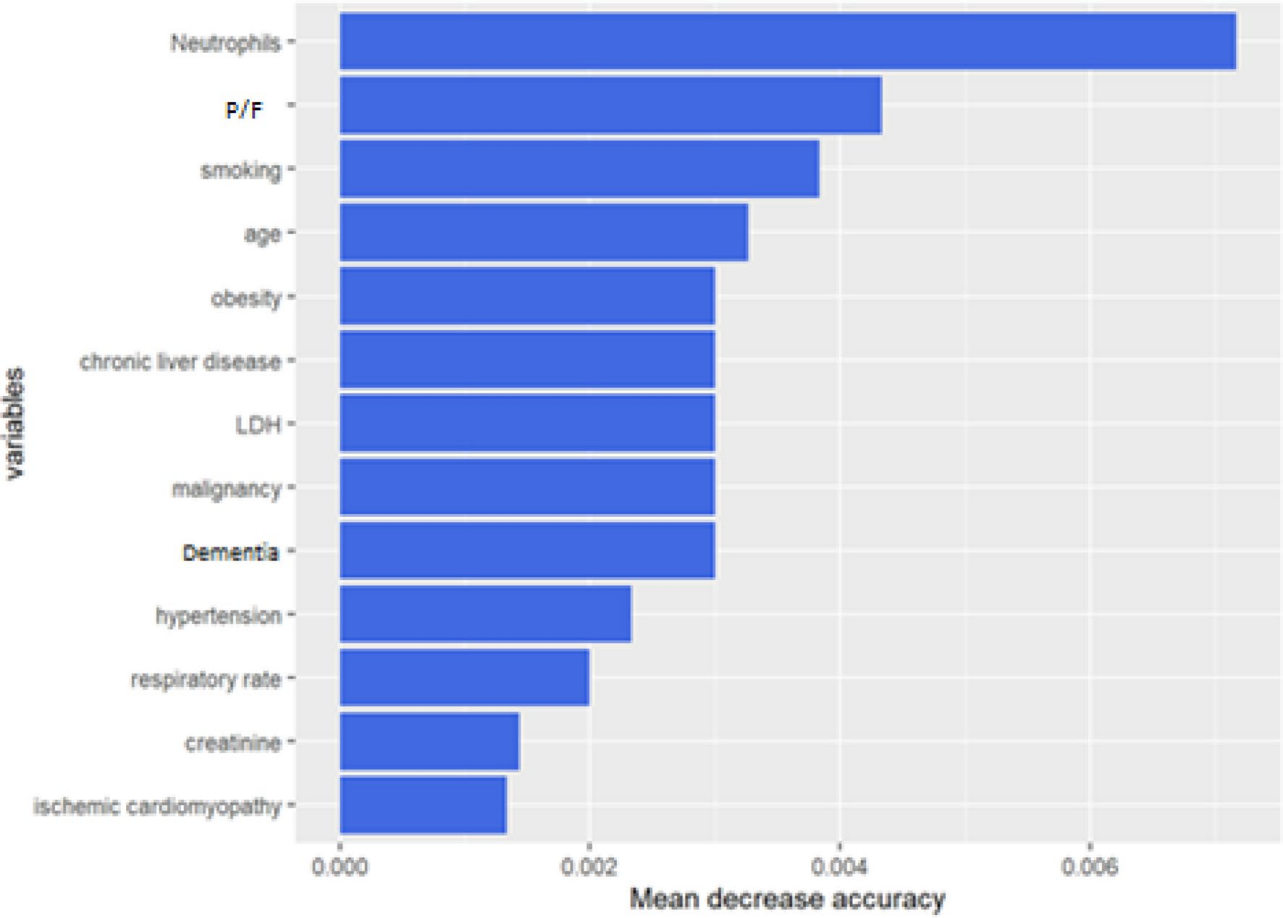
Random forest variable importance plot. The variables have been ranked in order of relevance in predicting in-hospital mortality. The importance measure considered for the analysis is the mean decrease in accuracy computed via Random Forest Classification Algorithm. The Random forest model accuracy is equal to 82.5% achieved with 1000 trees and 3 mtry.

The clinical predictor of mortality to be included in a multivariable logistic regression model have been identified via Random Forest feature selection (82.5% achieved accuracy with 1000 trees and 3 mtry). The variable importance measures for each predictor are represented in the Fig. 4.

As shown in Table 2, older age, smoking habit, obesity and the concomitant presence of active cancer turned out as independent predictors of mortality.

**Table 2:** Multivariable models.

Variable	OR	SE	*p*
**Panel 1 Multivariable logistic regression Set 1 (Harrel C = 0.79)**			
Age (58–80)	6.1226	0.38552	< 0.001
Active malignacy	4.6777	0.59049	0.009
Obesity	3.3966	0.43958	0.005
Smoking	2.7208	0.46454	0.031
**Panel 2 Multivariable logistic regression Set 2 (Harrel C = 0.78)**			
PF (285–137)	5.78	0.58486	0.003
Neutrophils (3.4–7.2)	1.0612	0.2744	0.829
Creatinine (0.73–1.14)	1.1881	0.15147	0.255
**Panel 2 Multivariable logistic regression Set 3 (Harrel C = 0.80)**			
Neutrophils (3.4–7.2)	1.209	0.33061	0.566
PF (285–137)	8.03	0.84283	0.013
Smoking	3.7057	0.97641	0.18
Age (58–80)	7.0326	0.92655	0.035
Dementia	1.4516	0.8434	0.659
Active malignancy	17.688	1.2803	0.025

Multivariable logistic regression estimates according to prediction Sets. Odds ratio (OR) with standard error (SE) and *p* values (*p*) have been reported. The .632 bootstrap (1000 resamples) Harrell-C statistics corrected for over-optimism is also reported.

### Laboratory predictors of mortality

To evaluate the association between laboratory variables at admission and prognosis, we considered a further subgroup of 256 patients, whose laboratory data are reported in Table 3.

**Table 3:** Laboratory characteristics of the study population.

	Total (N. 256)	Discharged (N. 161)	Dead (N. 55)	*p*
White blood cells (10^3^/ul)	6.5 [5.1–9.5]	6.3 [5.1–9.1]	7.5 [5.4–11.7]	0.07
Neutrophils (10^3^/ul)	4.6 [3.4–7.3]	4.5 [3.4–6.3]	6.4 [3.7–9.4]	0.01
Lymphocytes (10^3^/ul)	1.1 [0.8–1.5]	1.2 [0.9–1.6]	0.9 [0.6–1.3]	< 0.001
Hemoglobin (gr/dl)	13.6 [12.1–14.8]	13.9 [12.5–14.8]	13.3 [11.4–14.5]	0.14
Platelets (10^3^/ul)	188 [153–238]	197 [157–246]	159 [120–234]	0.01
Creatinine (mg/dl)	0.88 [0.73–1.14]	0.84 [0.72–1.06]	1.12 [0.86–1.77]	< 0.001
eGFR (ml/min)	83 [68–104]	90 [73–107]	70 [44–80]	< 0.001
ALT (UI/l)	31 [20–49]	32 [21–50]	30 [18–41]	0.19
LDH (UI/l)	623 [518–856]	601 [492–725]	706 [596–1168]	0.03
Potassium (mEq/l)	4.0 [3.7–4.4]	4.0 [3.7–4.4]	4.1 [3.8–4.7]	0.09
C-reactive protein (mg/dl)	7.1 [2.5–14.6]	4.5 [1.8–9.8]	14.4 [8.3–16.4]	0.01
D-dimer (µg/ml)	956 [541–1639]	919 [521–1593]	1356 [878–1688]	0.11
P/F ratio	223 [138–285]	246 [184–300]	126 [100–202]	< 0.001

*eGFR* estimated glomerular filtration rate, *ALT* alanine aminotransferases, *LDH* lactate dehydrogenases, *P/F* PaO_2_/FiO_2_ ratio, *COPD* chronic obstructive pulmonary disease.

PaO2/FiO2 (P/F) ratio was significantly lower in patients who died than in survivors, identifying a more severe respiratory failure at baseline (246 [184–300] vs. 126 [100–202]; < 0.001). In-hospital mortality was also associated with a higher neutrophil count and increased serum creatinine, C Reactive Protein (CRP), and Lactate Dehydrogenase (LDH). Finally, lower platelets and lymphocytes count were associated with mortality. In a multivariable model including the most relevant lab variables (identified via Random forest feature selection with the 78%% achieved accuracy with 1000 trees and 3 mtry), the P/F ratio was the only one to confirm its potential predictive role.

Finally, the P/F ratio was confirmed to predict mortality, along with age, in a further multivariable logistic regression model including demographic and clinical variables (Table 2). The predictors are selected via random forest mean decrease accuracy. The achieved accuracy is 78% with 1000 trees and 4 mtry.

The multivariable significant continuous predictors (Age and P/F ratio) achieved, separately, an acceptable AUC performance, comprised between 0.7 and 0.8 (Table 4), in agreement with the Hosmer’s indication^10^.

**Table 4:** Area under curve (AUC) estimation for the multivariable significant continuous death predictors.

	AUC	95% CI lower	95% CI upper
Age	0.75	0.71	0.80
PF	0.78	0.69	0.87

## Discussion

The pandemic diffusion of SARS-CoV-2 infection has suddenly thrown the international scientific community into uncertainty; we are facing a novel virus, with absolutely peculiar features, which is pushing all the National Health Systems under a level of pressure unexperienced before. One of the most challenging aspects of COVID-19 is its heterogeneity, with clinical pictures ranging from very mild to rapidly fatal; currently, it is unclear why some patients develop severe life-threatening disease, although possible pathogenetic mechanisms include a hyper-inflammatory reaction and a state of hypercoagulability^11,12^. Similarly, we are still unable to predict who will undergo clinical impairment and who, instead, will not. To make the situation even more confused, the clinical course and the prognosis of COVID-19 show huge differences worldwide. The fatality rate reported in South Europe and the United States of America (USA) is, for instance, significantly higher than in China or in North Europe^4^. It follows that findings obtained in a specific country might not be automatically extended to different geographic regions and that the depiction of national cohorts might contribute to explain this heterogeneity and to better stratify patients.

Although Italy has been hit hardly by the outbreak, especially in the North of the country, cohort studies describing the outcomes and the general features of COVID-19 patients in our geographic area are still lacking. This study was designed to fill this gap. According to our data, the in-hospital mortality in Northern Italy has been dauntingly high, close to 30%. In an outbreak, the infection fatality rate, i.e. the proportion of deaths among all the infected individuals, is commonly difficult to ascertain; this is particularly true for the infection by SARS-CoV-2, because of the presence of asymptomatic infected subjects prevents accurate estimates for the general population. While some countries applied a more stringent policy of intensive swabs testing, others were less prone to test subjects who are scarcely symptomatic. Moreover, the diagnostic accuracy of serological testing is still unclear. As we ignore the incidence of SARS-CoV-2 infection in the general population, we can only speculate that the fatality rate must be far lower than the CFR among hospitalized patients since hospital admission is limited to patients with a severe clinical picture. However, the difference between our data and those observed in Chinese cohorts is striking. In fact, in the earliest reports, the mortality among hospitalized patients has been estimated between 2.2 and 3.2%^13,14^. The ten-fold higher mortality observed in our cohort is probably related to a more severe clinical picture in our patients at hospital admission. Conceivably, the wider diffusion of the outbreak in Northern Italy led to admit to the hospital only those patients with a more severe clinical picture. Moreover, the first reports from China were based on populations with a median age significantly lower than that in our cohort, which might in part explain this discrepancy. In agreement with this hypothesis, in a recent retrospective Chinese study, the in-hospital mortality (28%) was similar to the one we observed, being older age, d-dimer levels and higher Sequential Organ Failure Assessment (SOFA) score on admission associated with higher odds of in-hospital death^15^.

To the best of our knowledge, no cohort studies are investigating in-hospital mortality in Italy. Recently, a collaborative initiative described the outcome and predictors of death in patients admitted to ICU in Lombardy, reporting a high mortality rate. In fact, among the 1581 patients with ICU disposition data available, 920 patients were still in the ICU at data censoring, 256 were discharged from the ICU, and 405 had died in the ICU^16^. However, there are no reports about a non-ICU setting. The largest cohort reported to date in Western Countries has been recently published by Richardson et al. and refers to 5700 COVID-19 inpatients in the New York City area^17^. Out of them, at data censoring, 2634 had completed their hospital stay, with a fatality rate of 21% (N = 553).

The mortality is, therefore, lower than in our cohort, but it should be kept in mind that USA and Italy are facing different phases of the epidemic; it can be argued that the fatality rate might be higher during the peak of the outbreak, which has been reached earlier in Italy, possibly explaining this discrepancy. However, the mortality among hospitalized patients in Western Countries is significantly higher than the one initially reported in China.

It is also interesting to remark the trend of mortality according to days from the clinical onset and hospital admission. Although the largest part of patients dies in the first days after hospital admission, there is a not negligible proportion of subjects who die later; actually, it is common experience of clinicians managing COVID-19 that besides those subjects showing a severe disease at hospital admission, there is a group of patients who clinically deteriorate after some days of hospital stay.

We then aimed to evaluate which clinical predictors might identify patients at higher risk of mortality. First of all, we considered only potential clinical predictors. According to our data age was confirmed as a strong independent predictor of mortality in all multivariable models. The impact of age is well defined in COVID-19 natural history and has been confirmed in any geographic region^13–15,17,18^. The real impact of gender is less clear even though there is a larger proportion of males than females dying because of COVID-19. According to the data reported by the Italian National Health System, males account for around 60% of total COVID-19 deaths, similar to what we reported^19^. This suggests a protective effect for the female gender; however, this gender difference seems not to exist in inpatients, in whom the age and the underlying comorbidities are more relevant^15^. Therefore, it is reasonable to postulate that females are protected against the development of severe COVID-19 infection, but once it is developed the risk of death is similar to males.

Among comorbidities, CAD, malignancies, chronic kidney disease, dementia, and hypertension were predictive of death at univariate analysis, but only malignancies and obesity fit into the multivariable analysis model. The latter variables seem to be, therefore, the most predictive comorbidities of death by COVID-19. The effect of CAD, chronic kidney disease, hypertension and dementia may be masked by the impact of age, being all age-related diseases. Cancer has already been described as an independent predictor of death. In particular, in a recent case–control study from an Italian group, COVID-19 patients mortality was significantly greater than that of a control group^20^. Similarly, obesity was already reported as an independent predictor of mortality in COVID-19, possibly because of the detrimental impact of fat-deriving cytokines on the clinical course of the disease^21,22^. Its predictive role is even more relevant considering the low prevalence of obesity in elderly, in our region (9.8% in Piedmont vs. 14.0% in Italy)^23^. The real impact of smoking is more debated and less defined; while some authors advocate its detrimental impact on the patient’s prognosis^24^, others conversely postulate a protective effect deriving from the down-regulation of Angiotensin Converting Enzyme-2 (ACE-2) expression in lungs^25^. According to our data, current smokers are at increased risk of mortality, although we should acknowledge that this information, as well as details about history of smoking, could not be retrieved for around one-hundred patients, making it challenging to accurately evaluate the effect of this risk factor. Therefore, further studies on larger cohorts are required to better elucidate this issue. However, our findings seem to be supported by a recent meta-analysis on 11590 COVID-19 patients, according to which smokers have higher odds of COVID-19 progression than never smokers^26^. Intriguingly, all the predictors of mortality that we identified are linked to prothrombotic status; this might be particularly relevant in COVID-19, in which arterial and venous thrombosis seems to play a pivotal role in determining a worse prognosis^27^.

Besides demographics and medical history, laboratory and clinical data may help in risk stratification; despite neutrophil count and creatinine predicted mortality at univariate analysis, in the multivariable model, the only laboratory predictor of death was P/F ratio. Respiratory failure severity seems to be a driving element in defining the prognosis; consistently, other relevant clinical predictors are dyspnea and a higher respiratory rate, whose association with a greater in-hospital mortality has already been reported^13,15^.

Our work contributed to identify a clinical phenotype of patients at higher mortality risk in a large Italian cohort of patients; in the near future and particularly in case of a further outbreak this might contribute to better identify those patients requiring a stricter follow-up and monitoring to detect early clinical deterioration.

Our study has several limitations; first of all, we miss clinical data of part of the population and we have laboratory data of only 256 patients. This is due to different concomitant causes, starting with the retrospective design of the study that prevented us to collect all the relevant data in a significant proportion of subjects. Moreover, and even more relevantly, this research was conducted during a National medical Emergency, which involved many clinicians in the management of a very high number of patients, making very hard to focus and dedicate time to research projects. The design of the study did not allow to accurately retrieve data able to stage the underlying diseases, potentially up or down-scoring the net effect of each comorbidity. Furthermore, as criteria for hospitalization of COVID-19 patients are different across different Institutions, an inclusion bias cannot be excluded in this regard. Finally, as this is an observational study, residual confounding factors may exist.

## Conclusions

In Italy, the COVID-19 outbreak determined a high in-hospital mortality, the main clinical predictors of which were age, current smoking, obesity, and a concomitant diagnosis of cancer. Among lab predictors, the P/F ratio, mirroring the severity of the respiratory failure, was the major factor associated with a severe prognosis.

## Supplementary information

Supplementary Table S1.

Supplementary Information.

## Data Availability

Raw data stored from patients records are pseudoanonymized and may be possibly linked to patients identity. Data can be accessed or reused upon request to the corresponding author who will share raw data after complete anonymization and clearance from competent authority (Comitato Interaziendale di Novara).
