# Supplementary material for: Fatality rate and predictors of mortality in an Italian cohort of hospitalized COVID-19 patients
Source: Sci Rep. 2020 Nov 26;10:20731. doi: 10.1038/s41598-020-77698-4 (PMC7692524; doi:10.1038/s41598-020-77698-4)
Supplement: Supplementary file 1 — Supplementary Table S1. [file 41598_2020_77698_MOESM1_ESM.docx]

**Fatality rate and predictors of mortality in an Italian cohort of hospitalized COVID-19 patients**

Mattia Bellan, MD, PhD^1,2^ Giuseppe Patti, MD^1,2^ Eyal Hayden, MD^1,2^ Danila Azzolina, MD, PhD^1^ Mario Pirisi, MD^1,2^ Antonio Acquaviva, MD^1,2^ Gianluca Aimaretti, MD, PhD^1,2^ Paolo Aluffi Valletti, MD^1,2^ Roberto Angilletta, MD^4^ Roberto Arioli, MD^1,2^ Gian Carlo Avanzi, MD^1,2^ Gianluca Avino, MD^1,2^ Piero Emilio Balbo, MD^2^ Giulia Baldon, MD^1,2^ Francesca Baorda, MD^1,3^ Emanuela Barbero, MD^1,2^  Alessio Baricich, MD^1,2^ Michela Barini, MD^2^ Francesco Barone-Adesi, MD, PhD^1^ Sofia Battistini, MD^1,2^ Michela Beltrame, MD^1,2^ Matteo Bertoli, MD^1,2^ Stephanie Bertolin, MD^1,2^ Marinella Bertolotti, MD^4^ Marta Betti, MD^4^ Flavio Bobbio, MD^2^ Paolo Boffano, MD^1,2^  Lucio Boglione, MD, PhD^1,3^ Silvio Borrè, MD^3^ Matteo Brucoli, MD^1,2^  Elisa Calzaducca, MD^1,2^ Edoardo Cammarata, MD^1,2^ Vincenzo Cantaluppi,^1,2^ Roberto Cantello, MD, PhD^1,2^ Andrea Capponi, MD^2^ Alessandro Carriero, MD^1,2^ Francesco Giuseppe Casciaro, MD^1,2^ Luigi Mario Castello, MD^1,2^ Federico Ceruti, MD^1,2^ Guido Chichino, MD^4^ Emilio Chirico, MD^1,2^ Carlo Cisari, MD^1,2^ Micol Giulia Cittone, MD^1,2^ Crizia Colombo, MD^1,2^ Cristoforo Comi, MD, PhD^1,3^ Eleonora Croce, MD^1,3^ Tommaso Daffara, MD^1,2^ Pietro Danna, MD^1,2^ Francesco Della Corte, MD^1,2^ Simona De Vecchi, MD^1,2^ Umberto Dianzani, MD, PhD^1,2^ Davide Di Benedetto, MD^1,2^ Elia Esposto, MD^1,2^ Fabrizio Faggiano, MD^1^ Zeno Falaschi, MD^1,2^ Daniela Ferrante, MD, PhD^1^ Alice Ferrero, MD^1,2^ Ileana Gagliardi, MD^1,2^ Gianluca Gaidano, MD, PhD^1,2^ Alessandra Galbiati, MD^1,2^ Silvia Gallo, MD^1,3^ Pietro Luigi Garavelli, MD^2^ Clara Ada Gardino, MD^1,2^ Massimiliano Garzaro, MD^1,2^  Maria Luisa Gastaldello, MD^1,2^ Francesco Gavelli, MD^1,2^ Alessandra Gennari, MD, PhD^1,2^ Greta Maria Giacomini, MD^1,2^ Irene Giacone, MD^1,3^ Valentina Giai Via, MD^1,2^ Francesca Giolitti, MD^1,2^ Laura Cristina Gironi, MD^1,2^ Carla Gramaglia, MD, PhD^1,2^ Leonardo Grisafi, MD^1,2^ Ilaria Inserra, MD^1,2^ Marco Invernizzi, MD, PhD^1,2^ Marco Krengli, MD^1,2^ Emanuela Labella, MD^1,2^ Irene Cecilia Landi, MD^1,2^ Raffaella Landi, MD^1,2^ Ilaria Leone, MD^1,2^ Veronica Lio, MD^1,2^ Luca Lorenzini, MD^1,2^ Antonio Maconi, MD^4^ Mario Malerba, MD^1,3^ Giulia Francesca Manfredi, MD^1,2^ Maria Martelli, MD^1,2^ Letizia Marzari, MD^1,2^ Paolo Marzullo, MD, PhD^1,2^ Marco Mennuni, MD^2^ Claudia Montabone, MD^1,3^ Umberto Morosini, MD^1,2^ Marco Mussa, MD^4^ Ilaria Nerici, MD^1,2^ Alessandro Nuzzo, MD^1,2^ Carlo Olivieri, MD^3^ Samuel Alberto Padelli, MD^1,3^ Massimiliano Panella, MD^1^ Andrea Parisini, MD^4^ Alessio Paschè, MD^1,2^ Alberto Pau, MD^1,2^  Anita Rebecca Pedrinelli, MD^1,2^ Ilaria Percivale, MD^1,2^ Roberta Re, MD^3^ Cristina Rigamonti, MD, PhD^1,2^ Eleonora Rizzi, MD^1,2^ Andrea Rognoni, MD^2^ Annalisa Roveta, MD^4^ Luigia Salamina, MD^2^ Matteo Santagostino, MD^2^ Massimo Saraceno, MD^1,2^ Paola Savoia, MD^1,2^ Marco Sciarra, MD^4^ Andrea Schimmenti, MD^4^ Lorenza Scotti, MD, PhD^1^ Enrico Spinoni, MD^1,2^ Carlo Smirne, MD, PhD^1,2^ Vanessa Tarantino, MD^1,2^ Paolo Amedeo Tillio, MD^1,3^ Rosanna Vaschetto, MD, PhD^1,2^ Veronica Vassia, MD^1,2^ Domenico Zagaria, MD^1,2^ Elisa Zavattaro, MD^2^ Patrizia Zeppegno, MD^1,2^ Francesca Zottarelli, MD^1,2^ and Pier Paolo Sainaghi, MD, PhD^1,2^.

1. Università del Piemonte Orientale UPO, Novara, Italy.

2 Azienda Ospedaliero Universitaria “Maggiore della Carita”, Novara, Italy.

3. Presidio Ospedaliero S. Andrea, ASL VC, Vercelli, Italy.

4. Azienda Ospedaliera SS. Antonio e Biagio e Cesare Arrigo, Alessandria, Italy.

*** Corresponding author:** Prof. Pier Paolo Sainaghi, Department of Translational Medicine, Università del Piemonte Orientale UPO, via Solaroli 17, Novara (NO), 28100, Italy. Tel.: +390321-3737512, email: [pierpaolo.sainaghi@med.uniupo.it](mailto:mattia.bellan@med.uniupo.it).

**Supplementary Table 1.** Univariate analysis of clinical and laboratory data. For abbreviation: OR, Odds Ratio, IQR, Interquartile range; P/F, PaO_2_/FiO_2_ ratio; ALT, alanine aminotransferases; LDH, lactate dehydrogenases;

| Variable | OR [IQR] | P |
| --- | --- | --- |
| Age | 1.08 [1.06-1.10] | <0.001 |
| P/F ratio | 0.99 [0.98-0.99] | <0.001 |
| Respiratory rate | 1.14 [1.08-1.20] | <0.001 |
| Dementia | 3.66 [2.02-6.63] | <0.001 |
| C-Reactive Protein | 1.13 [1.08-1.18] | <0.001 |
| Arterial hypertension | 2.67 [1.65-4.31] | <0.001 |
| Chronic Kidney disease | 4.28 [2.28-7.98] | <0.001 |
| Active Malignancy | 3.69 [1.77-7.71] | 0.001 |
| Creatinine | 2.22 [1.41-3.52] | 0.001 |
| Ischemic Cardiopathy | 2.43 [1.38-4.29] | 0.002 |
| Smoking | 2.28 [1.25-4.18] | 0.008 |
| Neutrophil count | 1.10 [1.02-1.18] | 0.016 |
| Diastolic arterial pressure | 0.98 [0.96-1.00] | 0.018 |
| Atrial fibrillation | 2.18 [1.14-4.18] | 0.019 |
| Potassium | 1.63 [1.07-2.49] | 0.024 |
| LDH | 1.00 [1.00-1.00] | 0.026 |
| Platelet count | 1.00 [0.99-1.00] | 0.048 |
| Heart rate | 1.01 [1.00-1.03] | 0.051 |
| Chronic liver disease | 2.93 [0.96-8.93] | 0.059 |
| Obesity | 1.75 [0.94-3.27] | 0.077 |
| ALT | 0.99 [0.98-1.00] | 0.100 |
| Hemoglobin | 0.89 [0.77-1.03] | 0.128 |
| Myalgia/fatigue | 0.70 [0.42-1.17] | 0.175 |
| COPD | 1.59 [0.79-3.23] | 0.197 |
| Diabetes | 1.36 [0.82-2.26] | 0.235 |
| Autoimmune disease | 2.07 [0.62-6.94] | 0.238 |
| Lymphocyte count | 1.05 [0.95-1.15] | 0.373 |
| Pre-existing interstitial lung disease | 1.77 [0.49-6.42] | 0.385 |
| Body temperature | 1.07 [0.87-1.32] | 0.521 |
| Gender | 0.89 [0.58-1.37] | 0.585 |
| Upper airway congestion | 0.73 [0.15-3.57] | 0.695 |
| D-dimer (541-1639 µg/ml) | 0.98 [0.87-1.1] | 0.724 |
| Systolic arterial pressure | 1.00 [0.99-1.01] | 0.942 |
