# Supplementary material for: Fatality rate and predictors of mortality in an Italian cohort of hospitalized COVID-19 patients
Source: Sci Rep. 2020 Nov 26;10:20731. doi: 10.1038/s41598-020-77698-4 (PMC7692524; doi:10.1038/s41598-020-77698-4)
Supplement: Supplementary file 2 — Supplementary Information. [file 41598_2020_77698_MOESM2_ESM.docx]

**Fatality rate and predictors of mortality in an Italian cohort of hospitalized COVID-19 patients**

Mattia Bellan, MD, PhD^1,2^ Giuseppe Patti, MD^1,2^ Eyal Hayden, MD^1,2^ Danila Azzolina, MD, PhD^1^ Mario Pirisi, MD^1,2^ Antonio Acquaviva, MD^1,2^ Gianluca Aimaretti, MD, PhD^1,2^ Paolo Aluffi Valletti, MD^1,2^ Roberto Angilletta, MD^4^ Roberto Arioli, MD^1,2^ Gian Carlo Avanzi, MD^1,2^ Gianluca Avino, MD^1,2^ Piero Emilio Balbo, MD^2^ Giulia Baldon, MD^1,2^ Francesca Baorda, MD^1,3^ Emanuela Barbero, MD^1,2^  Alessio Baricich, MD^1,2^ Michela Barini, MD^2^ Francesco Barone-Adesi, MD, PhD^1^ Sofia Battistini, MD^1,2^ Michela Beltrame, MD^1,2^ Matteo Bertoli, MD^1,2^ Stephanie Bertolin, MD^1,2^ Marinella Bertolotti, MD^4^ Marta Betti, MD^4^ Flavio Bobbio, MD^2^ Paolo Boffano, MD^1,2^  Lucio Boglione, MD, PhD^1,3^ Silvio Borrè, MD^3^ Matteo Brucoli, MD^1,2^  Elisa Calzaducca, MD^1,2^ Edoardo Cammarata, MD^1,2^ Vincenzo Cantaluppi,^1,2^ Roberto Cantello, MD, PhD^1,2^ Andrea Capponi, MD^2^ Alessandro Carriero, MD^1,2^ Francesco Giuseppe Casciaro, MD^1,2^ Luigi Mario Castello, MD^1,2^ Federico Ceruti, MD^1,2^ Guido Chichino, MD^4^ Emilio Chirico, MD^1,2^ Carlo Cisari, MD^1,2^ Micol Giulia Cittone, MD^1,2^ Crizia Colombo, MD^1,2^ Cristoforo Comi, MD, PhD^1,3^ Eleonora Croce, MD^1,3^ Tommaso Daffara, MD^1,2^ Pietro Danna, MD^1,2^ Francesco Della Corte, MD^1,2^ Simona De Vecchi, MD^1,2^ Umberto Dianzani, MD, PhD^1,2^ Davide Di Benedetto, MD^1,2^ Elia Esposto, MD^1,2^ Fabrizio Faggiano, MD^1^ Zeno Falaschi, MD^1,2^ Daniela Ferrante, MD, PhD^1^ Alice Ferrero, MD^1,2^ Ileana Gagliardi, MD^1,2^ Gianluca Gaidano, MD, PhD^1,2^ Alessandra Galbiati, MD^1,2^ Silvia Gallo, MD^1,3^ Pietro Luigi Garavelli, MD^2^ Clara Ada Gardino, MD^1,2^ Massimiliano Garzaro, MD^1,2^  Maria Luisa Gastaldello, MD^1,2^ Francesco Gavelli, MD^1,2^ Alessandra Gennari, MD, PhD^1,2^ Greta Maria Giacomini, MD^1,2^ Irene Giacone, MD^1,3^ Valentina Giai Via, MD^1,2^ Francesca Giolitti, MD^1,2^ Laura Cristina Gironi, MD^1,2^ Carla Gramaglia, MD, PhD^1,2^ Leonardo Grisafi, MD^1,2^ Ilaria Inserra, MD^1,2^ Marco Invernizzi, MD, PhD^1,2^ Marco Krengli, MD^1,2^ Emanuela Labella, MD^1,2^ Irene Cecilia Landi, MD^1,2^ Raffaella Landi, MD^1,2^ Ilaria Leone, MD^1,2^ Veronica Lio, MD^1,2^ Luca Lorenzini, MD^1,2^ Antonio Maconi, MD^4^ Mario Malerba, MD^1,3^ Giulia Francesca Manfredi, MD^1,2^ Maria Martelli, MD^1,2^ Letizia Marzari, MD^1,2^ Paolo Marzullo, MD, PhD^1,2^ Marco Mennuni, MD^2^ Claudia Montabone, MD^1,3^ Umberto Morosini, MD^1,2^ Marco Mussa, MD^4^ Ilaria Nerici, MD^1,2^ Alessandro Nuzzo, MD^1,2^ Carlo Olivieri, MD^3^ Samuel Alberto Padelli, MD^1,3^ Massimiliano Panella, MD^1^ Andrea Parisini, MD^4^ Alessio Paschè, MD^1,2^ Alberto Pau, MD^1,2^  Anita Rebecca Pedrinelli, MD^1,2^ Ilaria Percivale, MD^1,2^ Roberta Re, MD^3^ Cristina Rigamonti, MD, PhD^1,2^ Eleonora Rizzi, MD^1,2^ Andrea Rognoni, MD^2^ Annalisa Roveta, MD^4^ Luigia Salamina, MD^2^ Matteo Santagostino, MD^2^ Massimo Saraceno, MD^1,2^ Paola Savoia, MD^1,2^ Marco Sciarra, MD^4^ Andrea Schimmenti, MD^4^ Lorenza Scotti, MD, PhD^1^ Enrico Spinoni, MD^1,2^ Carlo Smirne, MD, PhD^1,2^ Vanessa Tarantino, MD^1,2^ Paolo Amedeo Tillio, MD^1,3^ Rosanna Vaschetto, MD, PhD^1,2^ Veronica Vassia, MD^1,2^ Domenico Zagaria, MD^1,2^ Elisa Zavattaro, MD^2^ Patrizia Zeppegno, MD^1,2^ Francesca Zottarelli, MD^1,2^ and Pier Paolo Sainaghi, MD, PhD^1,2^.

1. Università del Piemonte Orientale UPO, Novara, Italy.

2 Azienda Ospedaliero Universitaria “Maggiore della Carita”, Novara, Italy.

3. Presidio Ospedaliero S. Andrea, ASL VC, Vercelli, Italy.

4. Azienda Ospedaliera SS. Antonio e Biagio e Cesare Arrigo, Alessandria, Italy.

*** Corresponding author:** Prof. Pier Paolo Sainaghi, Department of Translational Medicine, Università del Piemonte Orientale UPO, via Solaroli 17, Novara (NO), 28100, Italy. Tel.: +390321-3737512, email: [pierpaolo.sainaghi@med.uniupo.it](mailto:mattia.bellan@med.uniupo.it).

**Acnowledgments:**

The present research has been perfomed by the COVID UPO Clinical Team a consortium that includes all the authors of the manuscript together to the following collaborators who have been involved in patients care and data generation: Giuseppe Aiosa,^4^ Andrea Airoldi,^2^ Ambra Barco,^2^ Olivia Bargiacchi,^2^ Simona Bazzano,^2^ Paola Berni,^2^ Bianca Bianchi,^2^ Sara Bianco,^2^ Stefano Biffi,^2^ Valeria Binda,^2^ Tatiana Bolgeo,^4^ Cesare Bolla,^4^ Valeria Bonato,^4^ Giacomo Bonizzoni,^4^ Alice Bragantini,^4^ Diego Brustia,^2^ Valentina Bullara,^2^ Michela Burlone,^2^ Fabio Brustia,^2^ Stefano Caccia,^2^ Anna Calareso,^3^ Gianmaria Cammarota,^2^ Laura Cancelliere,^2^ Roberto Carbone,^4^ Antonella Cassinari,^4^ Elisa Ceriani,^2^ Tiziana Cena,^2^ Elisa Clivati,^2^ Laura Collimedaglia^3^, Andrea Colombatto,^2^ Cristina Cornella,^2^ Martina Costanzo,^1,2^ Alessandro Croce,^1,2^ Carla De Benedittis,^1,2^ Stefania Delorenzi,^4^ Rosa Dionisio,^4^ Paolo Donato,^2^ Maria Esposito,^2^ Stefano Fangazio,^2^ Alessandro Feggi,^2^ Sara Ferrillo,^4^ Valentina Foci,^3^ Gian Paolo Fra,^2^ Claudio Gaggino,^4^ Eleonora Gambaro,^2^ Eleonora Gattoni,^2^ Luca Gattoni,^2^ Fabio Giacchero,^4^ Romina Gianfreda,^4^ Ailia Giubertoni,^2^ Lorenzo Grecu ,^4^ Francesca Grossi,^2^ Gabriele Guglielmetti,^2^ Stefania Guido,^2^ Giacomo Iannantuoni,^2^ Susanna Ingrao,^2^ Amalia Jona,^2^ ^2^ Elisa Lazzarich,^2^ Raffaella Lissandrin,^4^ Elisabetta Maduli,^2^ Federica Magnè,^4^ Eugenio Mantia,^4^ Debora Marangon,^2^ Maurilio Massara,^2^ Erica Matino,^2^ Maria Grazia Mauri,^2^ Mirta Menegatti,^2^ Roberta Moglia,^2^ Rossella Molinari,^2^ Stefania Morelli,^2^ Paola Morlino,^2^ Paola Naldi,^2^ Claudio Nebbiolo,^2^ Pinuccia Omodeo,^4^ Daniela Palmieri,^2^ Antonio Panero,^2^ Massimiliano Parodi,^4^ Filippo Patrucco,^1,3^ Roberta Pedrazzoli,^2^ Carolina Pelazza,^4^ Serena Penpa,^4^ Raffaella Perucca,^2^ Alice Pirovano,^1,2^ Sergio Pittau,^2^ Patrizia Pochetti,^3^ Federica Poletti,^2^ Biagio Polla,^4^ Paolo Prandi,^2^ Flavia Prodam,^1,2^ Pierluigi Prosperini,^2^ Alessia Puma,^2^ Marco Quaglia,^2^ Alberto Raie,^3^ Rachele Rapetti,^2^ Silvia Ravera ,^4^ Azzurra Re,^2^ Matia Reale,^4^ Antonella Rossati,^2^ Maura Rossi,^4^ Paola Rossi,^4^ Roberto Rostagno,^2^ Giulia Salomoni,^4^ Maria Teresa Samà,^2^ Eleonora Sarchi,^4^ Maddalena Sarcoli,^2^ Cristina Sarda,^4^ Ilaria Sguazzotti,^2^ Daniele Soddu,^1,2^ Daniele Sola,^2^ Paolo Stobbione,^4^ Monica Todoerti,^4^ Gian Carlo Vallese,^2^ Claudia Varrasi,^2^ Alessia Veia,^2^ Gian Luca Vignazia,^2^ Isabella Zanotti,^2^ Erika Zecca,^1,2^ Daniela Zichittella,^4^ Giuliana Zisa,^2^ Elisabetta Zoppis.^2^ The COVID UPO Clinical Team is represented by Prof. Pier Paolo Sainaghi.
